# MicroRNAs 143 and 150 in whole blood enable detection of T-cell immunoparalysis in sepsis

**DOI:** 10.1186/s10020-018-0056-z

**Published:** 2018-10-17

**Authors:** P Möhnle, S Hirschberger, L C Hinske, J Briegel, M Hübner, S Weis, G Dimopoulos, M Bauer, E J Giamarellos-Bourboulis, S Kreth

**Affiliations:** 1 Department of Anaesthesiology and Intensive Care Medicine, University Hospital, Ludwig Maximilian University (LMU), Marchioninistraße 15, 81377 Munich, Germany; 2 Walter-Brendel-Center of Experimental Medicine, Ludwig Maximilian University (LMU), Munich, Germany; 3 Department of Anaesthesiology and Intensive Care Medicine, Friedrich-Schiller University, Jena, Germany; 4 Center for Sepsis Control and Care, Jena University Hospital, Jena, Germany; 5 Center for Infectious Disease and Infection Control, Jena University Hospital, Jena, Germany; 6 2nd Department of Critical Care Medicine, ATTIKON University Hospital, National and Kapodistrian University of Athens, Athens, Greece; 7 4th Department of Internal Medicine, ATTIKON University Hospital, National and Kapodistrian University of Athens, Athens, Greece

**Keywords:** Sepsis, Immunoparalysis, T-cells, T cell exhaustion, miRNA, Biomarker

## Abstract

**Background:**

Currently, no suitable clinical marker for detection of septic immunosuppression is available. We aimed at identifying microRNAs that could serve as biomarkers of T-cell mediated immunoparalysis in sepsis.

**Methods:**

RNA was isolated from purified T-cells or from whole blood cells obtained from septic patients and healthy volunteers. Differentially regulated miRNAs were identified by miRNA Microarray (*n* = 7). Validation was performed via qPCR (*n* = 31).

**Results:**

T-cells of septic patients revealed characteristics of immunosuppression: Pro-inflammatory miR-150 and miR-342 were downregulated, whereas anti-inflammatory miR-15a, miR-16, miR-93, miR-143, miR-223 and miR-424 were upregulated. Assessment of T-cell effector status showed significantly reduced mRNA-levels of IL2, IL7R and ICOS, and increased levels of IL4, IL10 and TGF-β. The individual extent of immunosuppression differed markedly. MicroRNA-143, − 150 and − 223 independently indicated T-cell immunoparalysis and significantly correlated with patient’s IL7R-/ICOS-expression and SOFA-scores. In whole blood, composed of innate and adaptive immune cells, both traits of immunosuppression and hyperinflammation were detected. Importantly, miR-143 and miR-150 – both predominantly expressed in T-cells – retained strong power of discrimination also in whole blood samples.

**Conclusions:**

These findings suggest miR-143 and miR-150 as promising markers for detection of T-cell immunosuppression in whole blood and may help to develop new approaches for miRNA-based diagnostic in sepsis.

**Electronic supplementary material:**

The online version of this article (10.1186/s10020-018-0056-z) contains supplementary material, which is available to authorized users.

## Background

Sepsis has long been viewed as a disease with sequentially proceeding phases of hyperinflammation and immunoparalysis. Currently, it has become increasingly clear that sepsis is more a complex syndrome than a disease: Recent studies have indicated that states of hyperinflammation, largely driven by innate immune cells, and immunosuppression, mainly affecting adaptive immunity, can occur at any time, sequentially or even simultaneously (Boomer et al. 2014; Xiao et al. 2011). Immunoparalysis, however, has been identified as the major clinical problem leading to death in a large number of patients (Boomer et al. 2011). In this situation, effective tools for early detection and consecutive monitoring of immunosuppression are lacking, which reduces therapeutic.

success and hampers development of new strategies targeting immune dysfunction. Commonly used biomarkers in sepsis, e.g. C-reactive Protein, Interleukin-6, and Procalcitonin, lack sensitivity and specificity and cannot indicate immunosuppression (Samraj et al. 2013). Very recent attempts to assess an impaired immune status based on quantification of cell surface markers (HLA-DR3) or mRNAs (IL-10, CD74) have not yet made their way into clinical practice, as these strategies either rely on flow cytometric analysis, which is technically highly demanding, or on quantification of mRNA transcripts, which is always threatened by the mRNA’s intrinsic instability (Landelle et al. 2010; Peronnet et al. 2017). MiRNAs might bear the potential to fill this gap.

MicroRNAs are small non-coding RNAs acting as key regulators in gene expression networks, thus playing a pivotal role in almost all cellular processes (Bartel 2009). By base-pairing to the 3′-untranslated region (3’UTR) of their respective target genes, miRNAs posttranscriptionally repress gene expression (Bartel 2004). MicroRNAs display regulatory potential in a wide range of human diseases including cardiovascular conditions, degenerative processes, systemic inflammation and cancer (Pencheva and Tavazoie 2013; Hata 2013; Jung and Suh 2014; O'Connell et al. 2012; Hirschberger et al. 2018). In addition, identification and validation of miRNAs as biomarkers is an emerging field in medicine, given their tissue- and disease-specific expression and high stability even when released into the circulation (Weiland et al. 2012). In sepsis, recent publications reported altered expression of specific miRNAs. The authors suggested individual or sets of miRNAs as biomarkers to enable an early diagnosis, to differentiate different sepsis severity grades, and/or to predict survival (reviewed in (Kreth et al. 2017)). Results of these studies, however, were remarkably heterogeneous, and a consensus with respect to actually suitable biomarkers has not been reached so far. Disparate findings of these studies were most likely due to varying study aims, small sample sizes, and use of different sample types (either plasma/serum or whole blood or peripheral blood mononuclear cells) (Benz et al. 2016; Ho et al. 2016; Kingsley and Bhat 2017). The latter is of particular importance as different blood cell types exhibit highly specific transcriptomes and, naturally, differ considerably in miRNA expression profiles (Ecker et al. 2017; Leidinger et al. 2014; Palmer et al. 2006). Taking into consideration the fact that innate and adaptive immune cells may be regulated in a diametrically opposed way during sepsis (Boomer et al. 2014; Xiao et al. 2011; Cavaillon and Annane 2006; Tang et al. 2010), a more specific approach for the detection of immunosuppression is needed.

We hypothesized that specific miRNAs are capable to detect and to characterize sepsis-associated immunoparalysis. As particularly lymphocytes represent the suppressed adaptive immune response, we first set out to identify suitable miRNAs in T-cells and then – to facilitate clinical application – transferred our findings to whole blood samples. We here present a pilot study using miRNAs to specifically assess immunosuppression in sepsis.

## Methods

### Blood sampling

After obtaining informed consent, blood samples were withdrawn from healthy volunteers and from patients diagnosed with either sepsis or septic shock (by fulfilling the criteria SIRS + infection, according to the American College of Chest Physicians/Society of Critical Care Medicine consensus conference (Bone et al. 1992)). Retrospective evaluation showed that all patients were meeting the Sepsis-3 definitions (Singer et al. 2016). Patient analysis and microRNA evaluation was performed between 2011 and 2017, controls have been sampled from 2010 to 2016. For whole blood analysis a second, independent patient/ control group was analysed. The study protocol was approved by the Institutional Ethics Committees of the Ludwig-Maximilian-University Munich, Germany (No. 107–11 and No. 287–13; approved in 2006), of the University Hospital of Jena (No.2007–004333-42, local amendment for Munich University Hospital 2242–03/08), and by the Ethics Committee of ATTIKON University Hospital (approval 5/2008). Research was performed in accordance to the Declaration of Helsinki (ethical principles for medical research involving human subjects). Samples of sepsis patients were withdrawn immediately after diagnosis of sepsis and admission to the intensive care unit and before induction of an antibiotic and/or steroid treatment. History of malignant diseases, immunodeficiency, age younger than 18 years, and previous corticosteroid or antibiotic therapy were exclusion criteria. Patient characteristics are listed in Tables 1, 2, 3, 4 and 5.

**Table 1:** miRNA Microarray: Characteristics of Sepsis Patients

n	7
Gender (male/female)	3/4 (42.9%/ 57.1%)
Age, years (mean ± SD)	65.1 (± 13.4)
Septic Shock	4 (57.1%)
Sequential organ failure score (mean ± SD)	14.1 (± 3.5)
Nonsurvivors	3 (42.9%)

**Table 2:** T-cell samples: Characteristics of Sepsis Patients

n	31
Gender (male/female)	19/12 (61.2%/38.7%)
Age, years (mean ± SD)	57.2 (± 17.8)
Septic shock	14 (45.2%)
Sequential organ failure score (mean ± SD)	10.4 ± 5.4
Nonsurvivors	11 (35.5%)

**Table 3:** T-cell samples: Healthy controls

n	20
Gender (male/female)	12/8 (60%/40%)
Age, years (mean ± SD)	40.5 (± 5.5)

Healthy volunteers were all nonsmokers, without suspect of any acute or chronical disease, blood count and electrolytes within normal limits

**Table 4:** Whole blood samples: Characteristics of Sepsis Patients

n	20
Gender (male/female)	13/7 (65%/35%)
Age, years (mean ± SD)	76.4 ± 6.0
Septic shock	11 (55%)
APACHE II Score (mean ± SD)	23,4 ± 7,9
Nonsurvivors	10 (50%)

**Table 5:** Whole blood samples: Healthy controls

n	10
Gender (male/female)	6/4 (60%/40%)
Age, years (mean ± SD)	77,8 ± 7,7

Healthy volunteers were age-matched ambulatory patients before elective minor surgeries without suspect of severe chronical disease

### Blood cell isolation

After isolation of peripheral blood mononuclear cells (PBMCs) by density centrifugation (Histopaque 1077, Sigma-Aldrich, St. Louis, MO), T-cells were purified using an AutoMACS Pro Separator (Cat. # 130–092-545, Miltenyi Biotec, Bergisch-Gladbach, Germany) and magnetic cell separation (Pan T Cell Isolation Kit, Cat. # 130–096-535, Miltenyi Biotec, Bergisch Gladbach, Germany), as to the manufacturer’s recommendations. Cell number and viability were assessed using a ViCell analyzer (Beckman Coulter, Fullerton, CA). Only experiments exhibiting a cell viability of more than 90% were included in our analyses. By applying untouched negative selection, binding of isolation beads to T-cells and potential T-cell activation was avoided. Succesful T-cell isolation was confirmed by flow cytometry analysis using a FTIC anti-human CD3 antibody (Cat. # 344804, BioLegend, San Diego, CA, USA). whole blood analysis, PAXgene Blood RNA Tubes (PreAnalytiX, Hombrechtikon, Switzerland) were used according to the manufacturer’s instructions.

### RNA-isolation

Total RNA was isolated from primary T-cells using the mirVana miRNA Isolation Kit (Thermo Fisher Scientific, Waltham, MA, USA) with subsequent DNase treatment (Turbo DNase, Thermo Fisher Scientific, Waltham, MA, USA). Total RNA from whole blood samples was purified using the PAXgene Blood RNA Kit (PreAnalytiX, Hombrechtikon, Switzerland). The respective isolation procedures followed the manufacturer’s instructions. Quantity of total RNA was measured using a NanoDrop 2000 spectrophotometer (Thermo Fisher Scientific, Waltham, MA, USA), quality was verified by an Agilent 2100 Bioanalyzer. No differences in RNA quality/ quantity related to the age of RNA samples could be detected. Storage of RNA has been performed at − 80 °C. To further ensure stability of miRNA over time, the same RNA samples independently transcribed and analyzed in RT-qPCR in 2011, 2013 and 2017 have been depicted in Additional file 1: Figure S5.

### miRNA microarray

RNA from seven patients and from seven controls was used for miRNA microarray analysis (miRCURY LNA™ microRNA Array, Exiqon A/S, Vedbaek, Denmark), as to the manufacturer’s recommendations.

### Quantification of mRNA and miRNA expression

Expression of mRNAs and miRNAs was determined using a LightCycler 480 instrument (Roche Diagnostics, Mannheim, Germany) as described in (van der Heide et al. 2016). In all reactions, equal amounts of RNA were used (for miRNA transcription 6 ng of total RNA, for mRNA transcription 1000 ng of total RNA). TaqMan assays and specifications for qPCR primer and probes are given in Tables 6 and 7. Mean Target/Reference and Target/Reference standard deviation has been calculated for each miRNA/mRNA Target. Expression levels of septic patients are depicted relative to healthy control subjects. Determination of quantification cycles has been performed by the LightCycler software using the second derivative maximum method. Quantification cycle (Cq) cut-offs have been defined for miRNA (Cq 40) and mRNA (Cq 35) quantification. Cq values beyond cut-offs have been considered unspecific. For further validation of microRNA expression, both miR-143 and miR-150 in Pan T-cells and in whole blood cells have been assessed using additional internal controls U44 and U48; results are depicted in Additional file 2: Figure S1, Additional file 3: Figure S2, Additional file 4: Figure S3 and Additional file 5: Figure S4.

**Table 6:** TaqMan miRNA assays

Target	Assay Nr.
U44	001094
U47	001223
U48	001006
hsa-mir-15a	000389
hsa-mir-16	000391
hsa-mir-93	001090
hsa-mir-143	002249
hsa-mir-150	000473
hsa-mir-223	002295
hsa-mir-342	002260
hsa-mir-424	000604

**Table 7:** qPCR primer and probes

Target/ Probe/ primer direction	Primer sequence
SDHA #132 FW	5′-GAG GCA GGG TTT AAT ACA GCA-3′
SDHA #132 RV	5’-CCA GTT GTC CTC CTC CAT GT-3′
TBP #87 FW	5′-GAA CAT CAT GGA TCA GAA CAA CA-3′
TBP #87 RV	5′-ATA GGG ATT CCG GGA GTC AT-3′
IL-1β #41 FW	5’GAG GCA CAA GGC ACA ACA G-3‘
IL-1β #41 RV	5‘-CCA TGG CTG CTT CAG ACA C-3’
IL-2 #65 FW	5′-AAG TTT TAC ATG CCC AAG AAG G-3’
IL-2 #65 RV	5′-AAG TGA AAG TTT TTG CTT TGA GCT A-3’
IL-4 #38 FW	5’-TGC CTC ACA TTG TCA CTG C-3’
IL-4 #38 RV	5’-GCA CAT GCT AGC AGG AAG AAC-3’
IL-10 #67 FW	5’-TGC CTT CAG CAG AGT GAA GA-3’
IL-10 #67 RV	5’-GCA ACC CAG GTA ACC CTT AAA-3’
IL-7R #9 FW	5’-GCT TTT GAG GAC CCA GAT-3’
IL-7R #9 RV	5′ AGG CAC TTT ACC TCC ACG-3’
ICOS #2 FW	5’-TTC TGC TTG CGC ATT AAA GTT-3’
ICOS #2 RV	5’-CAT CTC ATA ATT GGC AGA ACC A-3’
TGF-β #31 FW	5‘-ACT ACT ACG CCA AGG AGG TCA C-3‘
TGF-β #31 RV	5‘-TGC TTG AAC TTG TCA TAG ATT TCG-3‘

*FW* forward primer, *RV* reverse primer; # probe number

### Statistical analysis

Student’s *t*-test or Mann-Whitney U tests, as appropriate, served for comparisons. Normal distribution was tested using the Kolmogorov-Smirnov test. The quantified array signals were background corrected (Normexp with offset value 10 - Convolution model described by Ritchie et al. (Ritchie et al. 2007)) and normalized using the global Lowess (LOcally WEighted Scatterplot Smoothing) regression algorithm. The obtained values were further analyzed using two-sided Student’s t-test and corrected via Benjamini-Hochberg False Discorvery Rate (Benjamini and Hochberg 1995). A false discovery value of less than 5% was considered significant.

Statistical analysis was performed using the statistical software R (Developement Core Team 2008) and GraphPad Prism 5.01 (GraphPad Software, Inc., USA). R 3.2.4 was used for predictive modeling and Area-under-the-ROC-curve (AUC) generation. All miRNAs that yielded significant expression differences in T-cells were selected for predictive modeling. R’s step-function was used for step-wise backward logistic regression on T-cell samples. AUCs were visualized and generated using the R-package pROC, with standard settings for bootstrapping. Vector artwork has been designed using Adobe Illustrator CS5.1 (Adobe Systems Inc., San Jose, CA, USA). Data are depicted as median, 25th and 75th percentile and outliers, if not stated otherwise. *P* values of less than 0.05 were defined as statistically significant (*p < 0.05; **p < 0.001).

## Results

### T-cells of septic patients exhibit a specific miRNA signature pointing to immunoparalysis

**Fig. 1:**
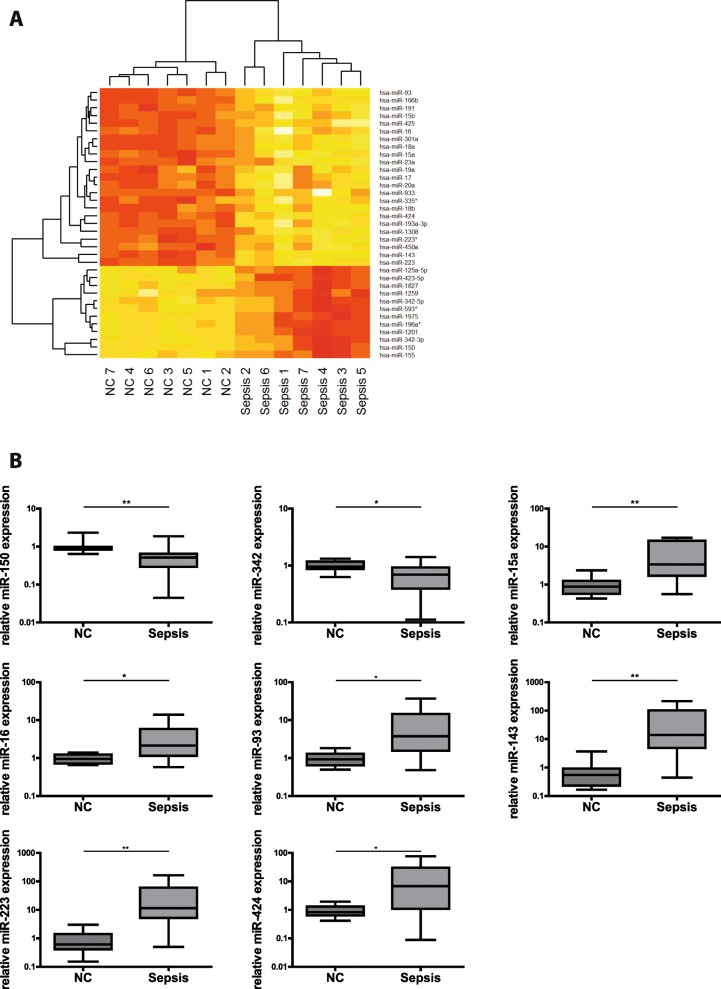
Differential expression of miRNA in septic T-cells. **a** MicroRNA Microarray analysis. Heat map showing the differentially expressed miRNAs in sepsis patients as compared to healthy controls, *n* = 7/7 (NC/Sepsis). RNA was isolated and miRNA array analysis was performed. Yellow colour indicates upregulation of miRNA expression, red colour indicates decreased miRNA levels. **b** MiRNAs in human Pan T-cells of septic patients as compared to healthy controls. Expression levels of miR-150, miR-342, miR-15a, miR-16, miR-93, miR-143, miR-223 and miR-424 in Pan T-cells of septic patients and healthy controls were measured by TaqMan miRNA assays relative to U47. Data are shown as median, 25th and 75th percentile and outliers, *n* = 10/20 (NC/Sepsis), performed in duplicates. Values represent expression relative to controls, *p < 0.05, **p < 0.001. Quantification cycle (Cq) values for the single miRNAs were in the range of 21 (NC) and 23 (Sepsis) for miR-150, 24 (NC) and 26 (Sepsis) for miR-342, 30 (NC) and 29 (Sepsis) for miR-15a, 23 (NC) and 22 (Sepsis) for miR-16, 30 (NC) and 28 (Sepsis) for miR-93, 34 (NC) and 29 (Sepsis) for miR-143, 26 (NC) and 22 (Sepsis) for miR-223, 36 (NC) and 34 (Sepsis) for miR-424, respectively

To investigate if miRNAs could serve as biomarkers indicative of immunoparalysis in sepsis, we performed a miRNA microarray using RNA isolated from T-cells of septic patients and from healthy individuals. As depicted in Fig. 1a, 35 miRNAs were identified as being differentially regulated in sepsis versus controls. Out of these, eight miRNAs revealed *p*-values < 0.01. Importantly, for all of them, a role in immunological processes has previously been described. The direction of regulation in our array analysis strongly points towards immunosuppression: miR-150 and miR-342 - both regulators of pro-inflammatory processes (Robertson et al. 2016; Roderburg et al. 2013) - were downregulated. Conversely, miR-15a, miR-16, miR-93, miR-143, miR-223 and miR-424 - all involved in anti-inflammatory signaling networks (Goodwin et al. 2015; Haneklaus et al. 2013; Honardoost et al. 2015; Liu et al. 2016; Zhao et al. 2014) - showed increased expression levels.

To validate our findings, we analyzed the expression levels of these miRNAs in a larger cohort of septic patients and healthy controls using TaqMan miRNA assays, which confirmed the initial array analysis (Fig. 1b): The expression of pro-inflammatory miR-150 and miR-342 was significantly reduced in septic T-cells, whereas anti-inflammatory miR-15a, miR-16, miR-93, miR-143, miR-223 and miR-424 showed markedly elevated levels (for fold induction analysis see also Additional file 6: Table S1). Taken together, our analysis of T-cells from sepsis patients identified a signature of eight differentially regulated miRNAs that - due to their biological functions - might indicate an immunosuppressive state.

### Cytokine expression profile of septic T-cells indicates immunoparalysis

**Fig. 2:**
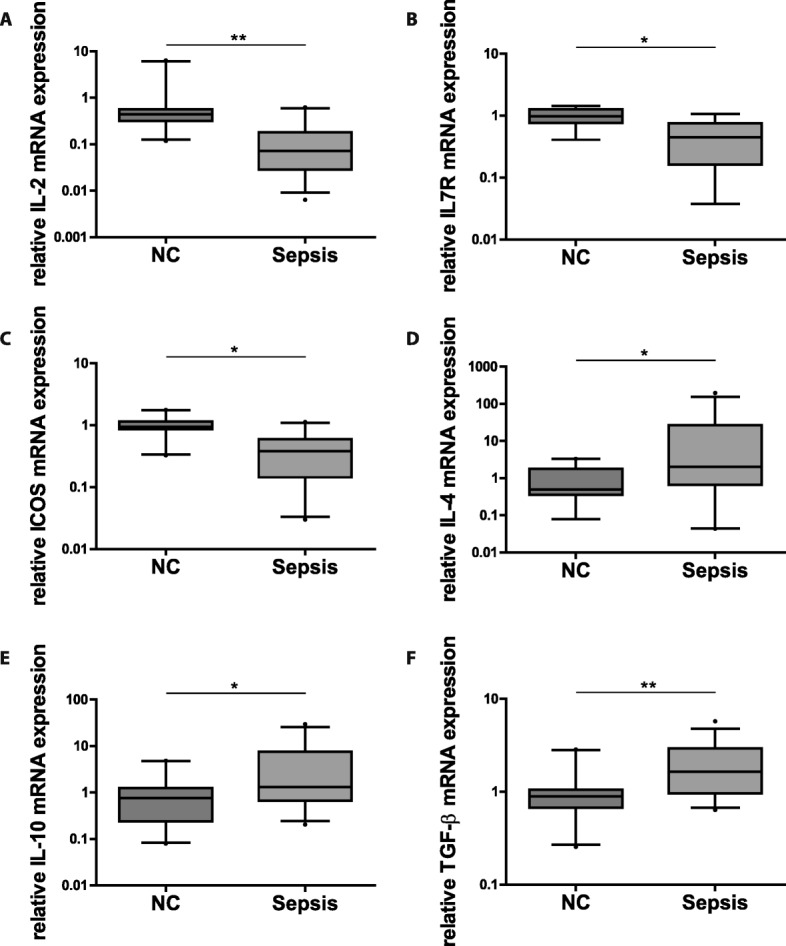
T-cell immunoparalysis in sepsis. Cytokine and immune receptor expression in T-cells of septic patients as compared to healthy controls. mRNA levels of (**a**) IL-2, (**b**) IL-7R (**c**) ICOS, (**d**) IL-4, (**e**) IL-10 and (**f**) TGF-ß in Pan T-cells of septic patients and healthy controls, respectively, were measured by qPCR relative to reference genes SDHA and TBP. Data are presented as median, 25th and 75th percentile and outliers, measurements were performed in duplicates. Values represent expression relative to controls, *p < 0.05, **p < 0.001. Quantification cycle (Cq) values for the single cytokines and receptors were in the range of 34 (NC) and 36 (Sepsis) for IL-2, 24 (NC) and 25 (Sepsis) for IL7R, 32 (NC) and 33 (Sepsis) for ICOS, 35 (NC) and 32 (Sepsis) for IL-4, 35 (NC) and 33 (Sepsis) for IL-10, 25 (NC) and 24 (Sepsis) for TGF-ß, respectively

To substantiate our assumption that the observed alterations in miRNA expression patterns are associated with immunoparalysis, we analyzed the expression of a set of characteristic pro- and anti-inflammatory cytokines as well as immune receptors relevant for T-cell immunity in the same set of T-cell samples. Compared to healthy controls, T-cells from septic patients showed significantly reduced expression levels of T-cell growth and survival factor interleukin 2 (IL-2), pro-inflammatory cytokine receptor interleukin 7 receptor (IL-7R) and inducible T-cell co-stimulator (ICOS) (Fig. 2a-c). Transcripts of T_H2_-cytokines interleukin 4 (IL-4) and interleukin 10 (IL-10) as well as T_reg_-differentiation promoting transforming growth factor beta (TGF-β) were markedly increased in sepsis samples (Fig. 2d-f). Collectively, we found expression patterns of cytokines, inflammatory mediators and immune receptors that suggest a state of immunoparalysis in T-cells of sepsis patients. These results were in line with the observed miRNA pattern.

### MicroRNA-223, microRNA-150 and microRNA-143 are markers of immunosuppression in T-cells

**Fig. 3:**
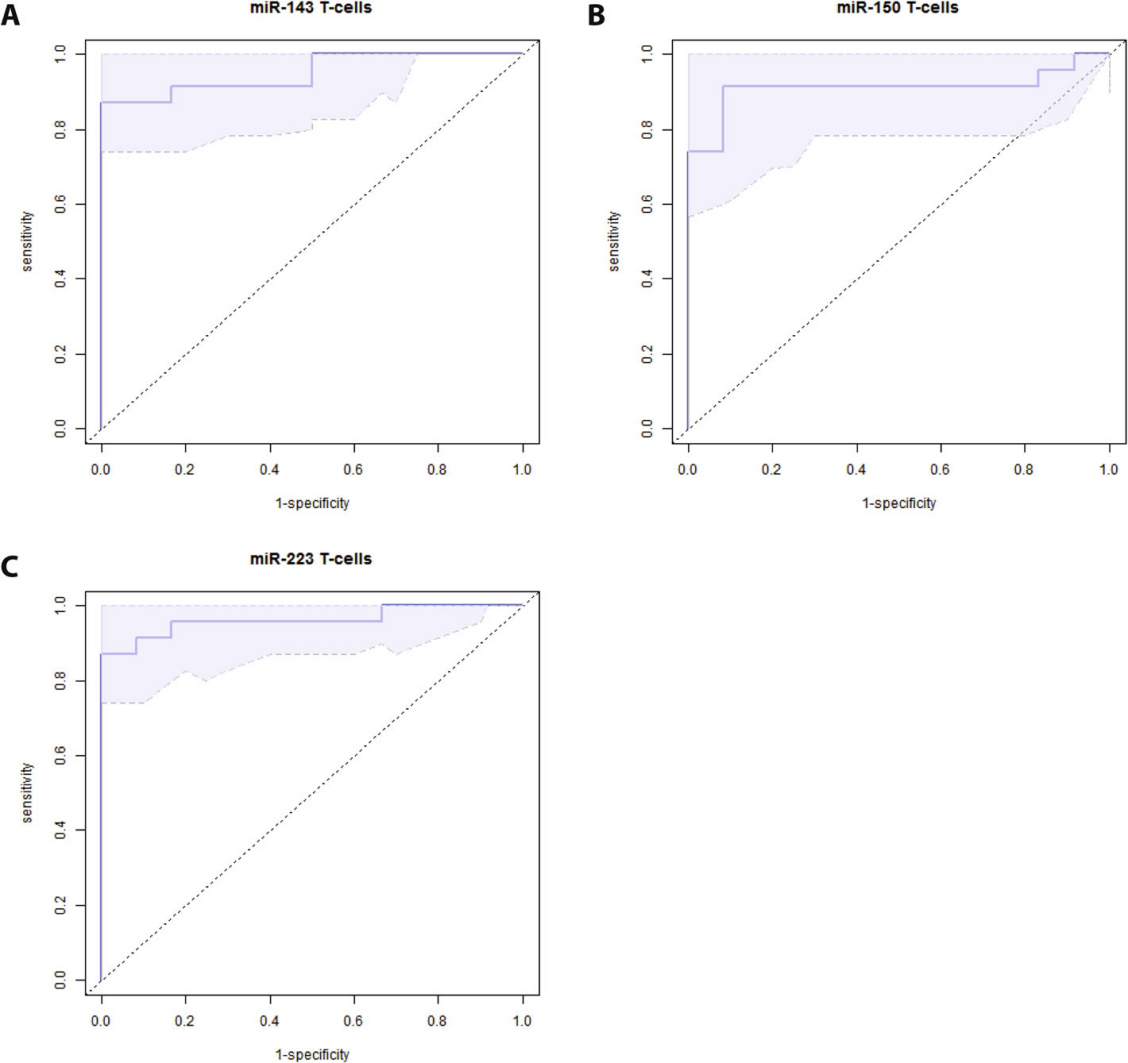
Discriminatory power of miR-143, miR-150 and miR-223 in T-cells. Predictive modeling and Area-under-the-ROC-curve generation. Candidate miRNAs were selected for predictive modeling: (**a**) miR-143, (**b**) miR-150 and (**c**) miR-223 in T-cells of septic patients as compared to healthy controls, *n* = 10/20 (NC/Sepsis). The shadowed areas denote the confidence interval for the area under the curve

To facilitate a potential clinical use, we next set out to evaluate, whether single miRNAs out of the signature might serve as surrogate marker for detection of sepsis-associated immunosuppression in T-cells. To this end, we used a variable selection procedure, focusing on those miRNAs exhibiting the highest significance with respect to differential expression in sepsis versus healthy controls (miR-223, miR-150 and miR-143). The best model for discrimination in T-cell samples displayed miR-223 with an excellent area under the curve (AUC) of 0.96 (95% CI: 0.9–1.0, Fig. 3). With AUCs of 0.91 (95% CI: 0.8–1.0) and 0.95 (95% CI: 0.88–1.0), miR-150 and miR-143 also revealed as very well performing markers. We next crosschecked whether these three miRNAs actually indicate impairment of adaptive immune functions and found highly significant correlations between the expression levels of these three miRNAs and the T-cell specific markers of immunosuppression IL7R and ICOS (miR-143: *r* = − 0.95 and − 0.78, *p* < 0.0005, miR-150: *r* = 0.87 and 0.66, *p* < 0.005, miR-223: r = − 0,78 and − 0,64 *p* < 0.05), and, importantly, with SOFA-scores (miR-143: 0.65, miR-150: − 0.7, miR-223: 0.57, *p* < 0.01; see also Table 8) Thus, we suggest that determination of these miRNAs in T-cells provides a useful method to assess immunoparalysis in sepsis.

**Table 8:** correlation analysis miR-143, −150, −223

	ICOS		IL7R		SOFA	
	r	*p*	r	*p*	r	*p*
miR-143	−0,78	< 0,0005	−0,95	< 0,0005	0,65	< 0,01
miR-150	0,66	< 0,005	0,87	< 0,005	−0,7	< 0,01
miR-223	−0,64	< 0,05	−0,78	< 0,05	0,57	< 0,01

### T-cell specific expression profiles are largely masked in whole blood samples

Purification of T-cells may be difficult to implement into a clinical setting. A widely used approach to obtain patient’s immune cell samples for gene expression analysis is the use of whole blood filter systems. While fast and easy to handle, they contain a mixture of cells of innate and adaptive immunity. It is therefore not clear, if T-cell related immunosuppression can be detected in these specimens.

**Fig. 4:**
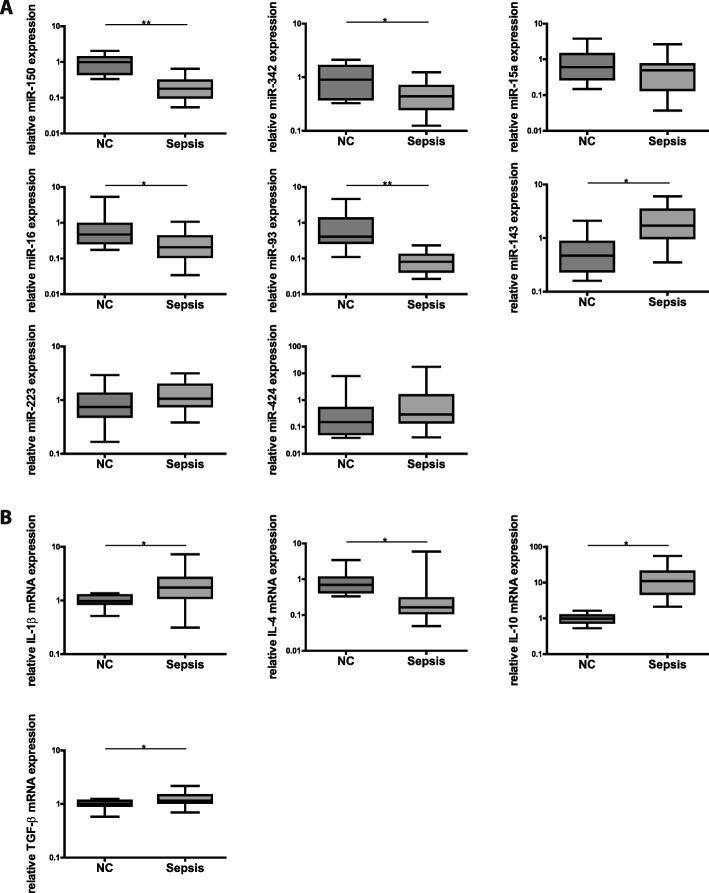
Simultaneous hyper- and hypoinflammation in whole blood of septic patients. **a** MiRNA expression in human whole blood obtained from septic patients as compared to healthy controls. Expression levels of miR-150, miR-342, miR-15a, miR-16, miR-93, miR-143, miR-223 and miR-424 were assessed by TaqMan miRNA assays relative to U47. Data are shown as median, 25th and 75th percentile and outliers, n = 10/20 (NC/Sepsis) performed in duplicates. Values represent expression relative to controls, *p < 0.05, **p < 0.001. Quantification cycle (Cq) values for the single miRNAs were in the range of 24 (NC) and 26 (Sepsis) for miR-150, 26 (NC) and 27 (Sepsis) for miR-342, 30 (NC) and 31 (Sepsis) for miR-15a, 21 (NC) and 23 (Sepsis) for miR-16, 26 (NC) and 29 (Sepsis) for miR-93, 34 (NC) and 33 (Sepsis) for miR-143, 21 (NC and Sepsis) for miR-223, 40 (NC) and 39 (Sepsis) for miR-424, respectively. **b** Cytokine expression in human whole blood obtained from septic patients as compared to healthy controls. mRNA levels of IL-4, IL-1β, IL-10 and TGF-β were measured by qPCR relative to reference genes SDHA and TBP. Data are shown as median, 25th and 75th percentile and outliers, n = 10/20 (NC/Sepsis) performed in duplicates. Values represent expression relative to controls, *p < 0.05, **p < 0.001. Quantification cycle (Cq) values for the single cytokines and receptors were in the range of 32 (NC) and 35 (Sepsis) for IL-4, 27 (NC and Sepsis) for IL-1β, 35 (NC) and 32 (Sepsis) for IL-10, 24 (NC) and 25 (Sepsis) for TGF-β, respectively

We thus aimed to test, whether those miRNAs identified as markers of T-cell immunosuppression might also perform sufficiently in whole blood samples. We first characterized PAXgene samples of 20 sepsis patients and ten healthy controls with respect to expression of the “immunosuppressive” miRNA signature and mRNA-levels of anti-inflammatory IL4, IL10, TGF-ß and pro-inflammatory “master cytokine” interleukin 1 beta (IL-1β). As shown in Fig. 4a, pro-inflammatory miRNAs miR-150 and miR-342 in Paxgene samples were also diminished in patients with sepsis as compared to healthy controls. Regarding anti-inflammatory miRNAs, only for miR-143 significant up-regulation could be detected, whereas miR-16 and miR-93 exhibited markedly reduced levels and miR-15a, miR-223 and miR-424 showed no relevant alterations at all. Analysis of cytokine expression (Fig. 4b) revealed significant upregulation of pro-inflammatory IL-1β but also of anti-inflammatory TGF-β. T_H2_-cytokines showed disparate results as well: IL-4 was downregulated, while IL-10 was upregulated.

Taken together, neither a distinct pro- nor anti-inflammatory miRNA/cytokine signature could be detected in whole blood samples. Since whole blood consists of a mixture of innate and adaptive immune cells - with neutrophils and lymphocytes accounting for a share of approximately 50% and 40%, respectively - miRNA and cytokine expression is likely to represent a net result of simultaneously occurring hyper- and hypoinflammation evoked by both innate and adaptive immunity.

### In whole blood samples, miR-143 and miR-150 can serve as markers of T-cell immunosuppression

Still aiming to use whole blood samples for detection of immunosuppression, we hypothesized that it might be a promising approach to focus on miRNAs that i.) are differentially regulated in the same direction in both sample types, and ii.) exhibit markedly lower expression levels in PAXgene samples than in T-cells. In these cases, polymorphonuclear cells (PMN) would not significantly contribute to the expression level of the respective miRNA in whole blood samples. Consequently, the effect seen in T-cells would – albeit diluted – be visible also in whole blood.

**Fig. 5:**
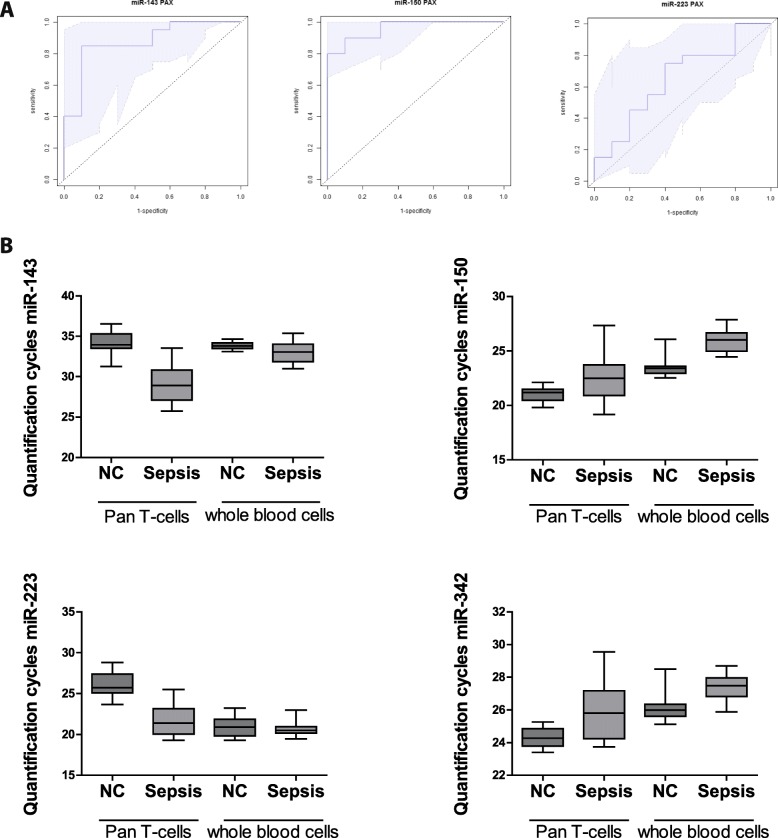
Discriminatory power of miR-143 and miR-150 in whole blood. **a** Predictive modeling and Area-under-the-ROC-curve generation. Candidate miRNAs were selected for predictive modeling: miR-143, miR-150 and miR-223 in whole blood samples of septic patients compared to healthy controls, *n* = 10/20 (NC/Sepsis). The shadowed areas denote the confidence interval for the area under the curve. **b** MiRNA quantification cycles of human CD4^+^ T-cells and whole blood cells. Samples of septic patients and healthy controls were measured, *n* = 10/20 (NC/Sepsis),performed in duplicates. Equal amounts of RNA were used to determine quantification cycles of miR-143, miR-150, miR-223 and miR-342 by TaqMan miRNA assay. Data are shown as median, 25th and 75th percentile and outliers

These criteria were met by miR-143, miR-150 and miR-342 (Figs. 4a and 5b). Using a variable selection procedure, miR-143 and miR-150 revealed a strong discriminative power (AUCs 0.88 (95% CI: 0.74–1.0) and 0.95 (95% CI: 0.9–1.0), Fig. 5a). MiR-223, the best performing marker in T-cells, was not differentially regulated and – as a typical innate miRNA – was found to be strongly expressed in PAXgene samples. Consequently, the resulting AUC was 0.66 (95% CI: 0.44–0.87, Fig. 5a), thus revealing miR-223 being not suitable as a biomarker for immunoparalysis in whole blood samples.

In summary, we suggest that whole blood analysis of miR-143 and miR-150 might be a promising strategy for the detection of T-cell immunosuppression in sepsis.

## Discussion

The dynamics of immune reactions during sepsis has long been regarded as a linear sequence of an initial hyperinflammatory phase followed by prolonged immunosuppression. Recent research, however, has revealed a more complex immunopathology: It is clear now that sepsis is a condition of constant immune dysfunction with alternating periods of pro- and anti-inflammatory predominance (Boomer et al. 2014), where hyperinflammation is mainly driven by innate immune cells, whereas immunoparalysis is a characteristic reaction of the adaptive immune system (Xiao et al. 2011; Hotchkiss et al. 2013). Immunosuppression has turned out the leading cause of death in sepsis (Boomer et al. 2011). While modern treatment concepts of sepsis are often capable to control hyperinflammation, therapy of immunoparalysis remains difficult and reliable methods allowing its early detection are lacking. In this situation, identification of miRNAs that are suitable to serve as biomarkers of a compromised immunity might be a promising approach.

We here identified a miRNA signature of eight differentially expressed miRNAs in T-cells of sepsis patients, which are indicative of sepsis-associated immunoparalysis. Of these miRNAs, miR-143 and miR-150 also performed well in whole blood samples: With AUCs of 0.88 (95% CI: 0.74–1.0) and 0.95 (95% CI: 0.9–1.0), respectively, they may even serve as surrogate biomarkers to assess septic T-cell immunoparalysis in a clinical setting, where the selection of T-cells is not feasible. These findings may open up new diagnostic perspectives for septic immune dysfunction.

MiRNAs offer unique features, which render them attractive as clinical biomarkers: They are expressed in disease-specific patterns, they are remarkably stable – even when released extracellularly – and they can easily be detected in virtually all tissues and body fluids (Benz et al. 2016). A considerable number of studies investigating miRNAs as biomarker in sepsis have been published in the last few years. In these studies, the expression of miRNAs was profiled in various tissue samples (plasma/serum/whole blood/purified blood cells) (Ho et al. 2016). Results, however, were heterogeneous with significant differences in expression profiles, e.g. miR-15/− 16 (Goodwin et al. 2015; Wang et al. 2012a; Wang et al. 2014a; Wang et al. 2015; Wang et al. 2012b; Wang et al. 2012c; Wang et al. 2014b), miR-146 (Wang et al. 2010; Wu et al. 2014; Wang et al. 2013), miR-150 (Roderburg et al. 2013; Vasilescu et al. 2009; Ma et al. 2013) and miR-223 (Wang et al. 2012a; Wang et al. 2015; Wang et al. 2012c; Wang et al. 2010). Thus, commonly accepted sepsis biomarkers are yet to be identified, in particular with respect to the discrimination between the hyperinflammatory and the immunoparalytic phase of the syndrome. As different types of human immune cells express cell-type-specific “miRNomes” (Leidinger et al. 2014; Ludwig et al. 2016; Wang et al. 2012d), evaluation of miRNAs as sepsis biomarkers in whole blood samples, serum, plasma or PBMCs is likely to yield mixtures of expression patterns. Moreover, a potential release of different miRNA signatures by acute or chronic co-morbidities may further hamper the diagnostic values of these approaches (Chen et al. 2008).

We aimed at avoiding such confounders by directly assessing the miRNA expression profile in T-cells using a whole transcriptome approach, and detected eight highly differentially expressed miRNAs, which were subsequently validated in a larger cohort of sepsis patients by qPCR. The observed miRNA expression strongly indicates a state of immunosuppression: MiR-150 and miR-342, both exerting proinflammatory action, were markedly downregulated. While miR-150 targets IL-10 and IL-18 in leukocytes, microRNA-342 contributes to broad host cell immunity against infection (Robertson et al. 2016). Also, low serum levels of miR-150 in critically ill patients have been associated with an unfavourable outcome, and miR-150has been discusssed as prognostic marker (leukocytes and plasma) and for discrimination between sepsis and SIRS (whole blood) (Roderburg et al. 2013; Vasilescu et al. 2009; Ma et al. 2013). The detected downregulation of both miRNAs thus may lead to a restriction of T-cell effector functions. Six further miRNAs – miR-15a, miR-16, miR-93, miR-143, miR-223 and miR-424, mainly exhibiting anti-inflammatory or anti-proliferative functions – were upregulated in septic T-cells. MiR-15a and miR-16 have been shown to downregulate NF-κB signaling pathways and to reduce pro-inflammatory cytokine production in T-cells via downregulation of CXCL10 (Goodwin et al. 2015; Liu et al. 2016). Moreover, they are known to exert pro-apoptotic effects in lymphocytes and granulocytes (Precone et al. 2013). MiR-93 is proposed to suppress T_H17_-cells (Honardoost et al. 2015), whereas miR-143 was reported to exhibit immunosuppressive functions via targeting COX-2 and TAK-1 (Zhao et al. 2014) and has been suggested as biomarker to distinguish between sepsis and SIRS (Han et al. 2016). (Zhao et al. 2014). MicroRNA-223 is interfering with several important inflammatory pathways, e.g. STAT1/3,NF-κB, and controls the NLRP3 inflammasome (Haneklaus et al. 2013). Collectively, upregulation of these miRNAs is likely to contribute to an immunosuppressive state.

Hallmarks of immunosuppression during sepsis have been defined in recent studies (Hamers et al. 2015): Lymphocytes express altered cytokine expression profiles with reduced levels of pro-inflammatory cytokines, enhanced production of anti-inflammatory cytokines (e.g. IL-10 and TGF-β), and receptor expression patterns favoring inhibitory receptors (Boomer et al. 2012; Gogos et al. 2000). Moreover, increased apoptosis rates, diminished IL-7 receptor expression and low levels of IL-2, indicating a reduced proliferative potential, have been found (Lang et al. 2005; Wherry and Kurachi 2015). In line with these data, we detected increased levels of anti-inflammatory cytokines IL-4, IL-10 and TGF-β as well as a reduction of IL-7R in isolated T-cells of sepsis patients, indicating a compromised T-cell immune status. Also, a substantial loss of IL-2 mRNA and diminished levels of T-cell inflammatory response regulation factor ICOS clearly suggest reduced survival and activity of effector T-cells (Boomer et al. 2014; Hotchkiss et al. 2013; Wikenheiser and Stumhofer 2016). Thus, in all sepsis patients, signs of immunoparalysis within the adaptive immunity could be detected. The extent, however, was inter-individually different and quantification of immunosuppression may provide valuable information to assess the actual state of the individual patient - also during the course of the disease - and to define optimal treatment strategies. In this dynamic situation, our miRNA signature could be a fast and reliable tool.

As requirement for simultaneous quantification of eight miRNAs might impede the clinical use of our approach, we next aimed at identifying key markers of immunosuppression. Indeed, miR-223, miR-143 and miR-150 exhibited an outstanding predictive power with AUCs of 0.96, 0.95 and 0.90. Importantly, these miRNAs significantly correlated with T-cell specific markers of immunosuppression and with SOFA scores. Therefore, all three miRNAs might be potential candidates to evaluate T-cell associated immunoparalysis in sepsis.

Even when limited to measurement of single miRNAs with sufficient discriminatory power, a T-cell-based approach may not be feasible in a clinical routine situation. In this setting, the commonly applied method is the use of filter systems, enabling a fast and easy-to-handle approach to obtain patient’s blood samples for gene expression analyses. It has to be kept in mind, however, that these filters retain a mixture of both innate and adaptive immune cells (with PMN, T-cells, and Monocytes making up for the largest shares of cells with 50%, 40%, and 10%) and it therefore is not clear, whether T-cell immunoparalysis is assessable in these samples. In our analyses, indeed, cytokine and miRNA expression profiles exhibited signs of both immune activation and suppression, which is likely to represent the net result of simultaneously occurring but divergent activation patterns of innate and adaptive immune cells, thereby masking T-cell specific effects. These findings are in line with previous studies showing that human leukocytes during sepsis do not express distinct pro- or anti-inflammatory profiles (Tang et al. 2010). Notwithstanding that, we assumed that innate immune cells would not significantly impair the diagnostic use of T-cell specific miRNAs in whole blood samples, if the expression levels of the respective miRNAs in PMN and Monocytes were markedly lower compared to T-cells. Indeed, we identified miR-143 and miR-150 as extremely well performing markers indicative of T-cell immunosuppression not only in T-cells but also in PAXgene samples: They were differentially regulated in the same direction in both sample types, they exhibited low expression levels in innate cells and - after applying a variable selection procedure - they showed excellent discriminatory power with AUCs of 0.88 and 0.95, respectively, also in PAXgene samples. Nevertheless, biases caused by larger shifts in lymphocyte numbers may occur when using PAXgene. As we included both immunosuppressive (upregulated) miR-143 and proinflammatory (downregulated) miR-150 into our analyses, such distortions could easily be detected. In our series, however, this phenomenon was not observed.

Medical and ethical restriction have limited the spectrum of possible molecular analyses of this pilot study:

1. We could only analyze mRNA and miRNA expression levels, additional analysis of protein levels or quantification of T-cell surface markers was not possible. However, as reported in a recent study, mRNA levels of the most relevant cytokines in activated T cells are translated into comparable protein levels (Mohnle et al. 2015).
2. We were only able to investigate Pan T-cells, which did not allow specifying septic T-cell immunoparalysis with respect to T-cell subsets. However, relatively stable CD4/CD8 ratios and unaltered CD4+ proportions in peripheral blood during sepsis have been described in several studies (Francois et al. 2018; Inoue et al. 2014).
3. Both patient groups differ in age, since the samples have been provided from ICUs of different countries and with different clinical focus. However, we analyzed the study cohorts independently with respect to the validity of our T cell miRNA markers. Remarkably, despite different age-distributions, both miR-143 and miR-150 performed comparably well in either group thus indicating age-independency.
4. Sampling of patients was performed over a long course of time. However, as microRNA is known for its outstanding stability (Jung et al. 2010; Mraz et al. 2009), bias due to sample degradation is unlikely. Furthermore, storage has been performed properly, RNA analysis showed no major differences in quality or quantity related to the age of RNA samples and additional RT-qPCR analyses revealed no significant alterations in microRNA level over time (Additional file 1: Figure S5).

## Conclusions

Efficient therapeutic interventions in septic patients are currently hampered by a lack of reliable biomarkers for diagnosis of sepsis-associated immune dysfunction. Our pilot study identifies miR-143 and miR-150 as candidate markers for detection of T-cell immunosuppression and thus contributes to the development of innovative strategies using miRNAs as biomarkers of a compromised immune status during sepsis. Importantly, these markers can be determined in whole blood samples, which facilitates a future clinical use. Large-scale, multicenter, prospective evaluations are now required to further elaborate our diagnostic approach and to enable its implementation into clinical routine.

## Additional files

Additional file 1: **Figure S5.** U47 Quantification cycles for samples analyzed in 2011, 2013 and 2017. Quantification cycles of U47 in (A) healthy controls and (B) septic patients were measured by TaqMan miRNA assays. The same RNA samples have been used for independent transcription and RT-qPCR analysis in the years 2011, 2013 and 2017, all experiments performed in duplicates. (EPS 1313 kb)

Additional file 2: **Figure S1.** Expression of microRNA-143 in T-cell samples using additional internal controls. Expression levels of miR-143 in Pan T-cells of septic patients and healthy controls were measured by TaqMan miRNA assays relative to (A) U44, (B) U47, (C) U48 and (D) U44/U47/U48. Data are shown as median, 25th and 75th percentile and outliers, *n* = 5/10 (NC/Sepsis), performed in duplicates. Values represent expression relative to controls, *p < 0.05, **p < 0.001. (EPS 747 kb)

Additional file 3: **Figure S2.** Expression of microRNA-150 in T-cell samples using additional internal controls. Expression levels of miR-150 in Pan T-cells of septic patients and healthy controls were measured by TaqMan miRNA assays relative to (A) U44, (B) U47, (C) U48 and (D) U44/U47/U48. Data are shown as median, 25th and 75th percentile and outliers, *n* = 5/10 (NC/Sepsis), performed in duplicates. Values represent expression relative to controls, *p < 0.05, **p < 0.001. (EPS 749 kb)

Additional file 4: **Figure S3.** Expression of microRNA-143 in whole blood samples using additional internal controls. Expression levels of miR-143 in whole blood cells of septic patients and healthy controls were measured by TaqMan miRNA assays relative to (A) U44, (B) U47, (C) U48 and (D) U44/U47/U48. Data are shown as median, 25th and 75th percentile and outliers, n = 5/10 (NC/Sepsis), performed in duplicates. Values represent expression relative to controls, *p < 0.05, **p < 0.001. (EPS 746 kb)

Additional file 5: **Figure S4.** Expression of microRNA-150 in whole blood samples using additional internal controls. Expression levels of miR-150 in whole blood cells of septic patients and healthy controls were measured by TaqMan miRNA assays relative to (A) U44, (B) U47, (C) U48 and (D) U44/U47/U48. Data are shown as median, 25th and 75th percentile and outliers, *n* = 5/10 (NC/Sepsis), performed in duplicates. Values represent expression relative to controls, *p < 0.05, **p < 0.001. (EPS 734 kb)

Additional file 6: **Table S1.** Fold difference miRNA expression in T-cells of septic patients compared to healthy controls. **Table S2.** Positive and negative predictive values for miR-143/− 150/ -223. **Table S3.** Benjamini-Hochberg correction: *p*-value and false discovery rate. (DOCX 19 kb)
