# Supplementary material for: MicroRNAs 143 and 150 in whole blood enable detection of T-cell immunoparalysis in sepsis
Source: Mol Med. 2018 Oct 17;24:54. doi: 10.1186/s10020-018-0056-z (PMC6191918; doi:10.1186/s10020-018-0056-z)
Supplement: Supplementary file 6 — Table S1. Fold difference miRNA expression in T-cells of septic patients compared to healthy controls. Table S2. Positive and negative predictive values for miR-143/− 150/ -223. Table S3. Benjamini-Hochberg correction: p-value and false discovery rate. (DOCX 19 kb) [file 10020_2018_56_MOESM6_ESM.docx]

Additional file 6: **Table S1.** Fold difference miRNA expression in T-cells of septic patients compared to healthy controls

|  | **NC** | | **Sepsis** | |
| --- | --- | --- | --- | --- |
| **microRNA** | **Mean** | **SD** | **Mean** | **SD** |
| **miR-150** | 1,0 | 0,438 | 0,527 | 0,385 |
| **miR-342** | 1,0 | 0,22 | 0,704 | 0,371 |
| **miR-15a** | 1,0 | 0,572 | 6,88 | 6,61 |
| **miR-16** | 1,0 | 0,27 | 3,66 | 3,41 |
| **miR-93** | 1,0 | 0,415 | 8,59 | 9,43 |
| **miR-143** | 1,0 | 1,22 | 58,50 | 68,30 |
| **miR-223** | 1,0 | 0,955 | 36,60 | 43,80 |
| **miR-424** | 1,0 | 0,45 | 21,20 | 19,30 |

**Table S2**. Positive and negative predictive values for miR-143/-150/ -223

| **miRNA** | **sample** | **sensitivity** | **specificity** | **PPV** | **NPV** | **% septic patients** |
| --- | --- | --- | --- | --- | --- | --- |
| **miR-223** | T-cells | 1.0 | 0.87 | 0.8 | 1.0 | 67 (23 out of 35) |
| **miR-150** | T-cells | 0.92 | 0.91 | 0.85 | 0.95 |  |
| **miR-143** | T-cells | 1.0 | 0.87 | 0.8 | 1.0 |  |
| **miR-223** | whole blood | 0.6 | 0.75 | 0.55 | 0.79 | 66 (20 out of 30) |
| **miR-150** | whole blood | 0.9 | 0.9 | 0.82 | 0.95 |  |
| **miR-143** | whole blood | 0.9 | 0.85 | 0.75 | 0.94 |  |

Cutoff determined using Youden's index (maximum sum of sensitivity + specificity). Positive and negative predictive values change with prevalence of sepsis and would therefore need to be recalculated to be applied.

**Table S3**. Benjamini-Hochberg correction: p-value and false discovery rate

| **miRNA** | **p-value** | **FDR** |
| --- | --- | --- |
| **hsa-miR-143** | 1,62494000E-07 | 7,71846500E-06 |
| **hsa-miR-424** | 1,46118000E-07 | 7,71846500E-06 |
| **hsa-miR-93** | 2,86506000E-07 | 9,07269000E-06 |
| **hsa-miR-223*** | 5,04946000E-07 | 1,17672890E-05 |
| **hsa-miR-106b** | 6,19331000E-07 | 1,17672890E-05 |
| **hsa-miR-223** | 9,45374000E-07 | 1,49684217E-05 |
| **hsa-miR-423-5p** | 2,17401000E-06 | 2,95044214E-05 |
| **hsa-miR-18a** | 3,66879000E-06 | 4,35668813E-05 |
| **hsa-miR-301a** | 1,07209000E-05 | 1,06827500E-04 |
| **hsa-miR-1201** | 1,12450000E-05 | 1,06827500E-04 |
| **hsa-miR-191** | 1,34114000E-05 | 1,07140269E-04 |
| **hsa-miR-15a** | 1,46613000E-05 | 1,07140269E-04 |
| **hsa-miR-1308** | 1,42072000E-05 | 1,07140269E-04 |
| **hsa-miR-1827** | 2,90868000E-05 | 1,97374714E-04 |
| **hsa-miR-196a*** | 3,93107000E-05 | 2,48967767E-04 |
| **hsa-miR-1975** | 6,83343000E-05 | 3,95664382E-04 |
| **hsa-miR-425** | 8,81680000E-05 | 4,29806600E-04 |
| **hsa-miR-18b** | 9,04856000E-05 | 4,29806600E-04 |
| **hsa-miR-15b** | 1,08738000E-04 | 4,91910000E-04 |
| **hsa-miR-193a-3p** | 1,16182000E-04 | 5,01695000E-04 |
| **hsa-miR-155** | 1,42259000E-04 | 5,63108542E-04 |
| **hsa-miR-125a-5p** | 1,36597000E-04 | 5,63108542E-04 |
| **hsa-miR-17** | 1,93569000E-04 | 7,35562200E-04 |
| **hsa-miR-450a** | 5,18363000E-04 | 1,60814774E-03 |
| **hsa-miR-23a** | 5,10034000E-04 | 1,60814774E-03 |
| **hsa-miR-19a** | 4,93817000E-04 | 1,60814774E-03 |
| **hsa-miR-593*** | 5,24764000E-04 | 1,60814774E-03 |
| **hsa-miR-342-3p** | 5,45580000E-04 | 1,61969063E-03 |
| **hsa-miR-16** | 6,62121000E-04 | 1,90610591E-03 |
| **hsa-miR-342-5p** | 7,22984000E-04 | 1,96238514E-03 |
| **hsa-miR-150** | 8,81330000E-04 | 2,23017615E-03 |
| **hsa-miR-335*** | 9,09720000E-04 | 2,23017615E-03 |
| **hsa-miR-20a** | 9,15546000E-04 | 2,23017615E-03 |
| **hsa-miR-933** | 9,60203000E-04 | 2,28048213E-03 |
| **hsa-miR-1259** | 9,89857000E-04 | 2,29357110E-03 |
| **hsa-miR-32*** | 1,15735600E-03 | 2,61782905E-03 |
| **hsa-miR-451** | 1,79845500E-03 | 3,71420054E-03 |
| **hsa-miR-24** | 2,09425200E-03 | 4,23306255E-03 |
| **hsa-miR-29a*** | 3,06950400E-03 | 5,83205760E-03 |
| **hsa-miR-23b** | 3,64236000E-03 | 6,78478824E-03 |
| **hsa-miR-29b-1*** | 4,61958500E-03 | 8,43962644E-03 |
| **hsa-miR-744** | 4,93811000E-03 | 8,85132925E-03 |
| **hsa-miR-185** | 7,02390200E-03 | 1,23568646E-02 |
| **hsa-miR-1274a** | 8,49561200E-03 | 1,44193952E-02 |
| **hsa-miR-1297** | 9,10713900E-03 | 1,51785650E-02 |
| **hsa-miR-25** | 1,03710140E-02 | 1,66990903E-02 |
| **hsa-miR-155*** | 1,27542700E-02 | 1,95428331E-02 |
| **hsa-miR-339-5p** | 1,26855100E-02 | 1,95428331E-02 |
| **hsa-miR-103** | 1,44499650E-02 | 2,17896298E-02 |
| **hsa-miR-1973** | 2,15298120E-02 | 3,19583147E-02 |
| **hsa-miR-106a** | 2,58945210E-02 | 3,72724166E-02 |
| **hsa-miR-186** | 3,30287780E-02 | 4,48506061E-02 |
| **hsa-miR-132*** | 3,53102280E-02 | 4,72460797E-02 |
